# Diet and *SIRT1* Genotype Interact to Modulate Aging-Related Processes in Patients with Coronary Heart Disease: From the CORDIOPREV Study

**DOI:** 10.3390/nu14183789

**Published:** 2022-09-14

**Authors:** Cristina Hidalgo-Moyano, Oriol Alberto Rangel-Zuñiga, Francisco Gomez-Delgado, Juan F. Alcala-Diaz, Fernando Rodriguez-Cantalejo, Elena M. Yubero-Serrano, Jose D. Torres-Peña, Antonio P. Arenas-de Larriva, Antonio Camargo, Pablo Perez-Martinez, Jose Lopez-Miranda, Javier Delgado-Lista

**Affiliations:** 1 Lipids and Atherosclerosis Unit, Department of Internal Medicine, Reina Sofia University Hospital, 14004 Córdoba, Spain; 2 Department of Medical and Surgical Sciences, University of Córdoba, 14004 Córdoba, Spain; 3 Maimónides Biomedical Research Institute of Córdoba (IMIBIC), 14004 Córdoba, Spain; 4 CIBER Fisiopatologia de la Obesidad y la Nutricion (CIBEROBN), Instituto de Salud Carlos III, 28029 Madrid, Spain; 5 Internal Medicine Unit, Jaen University Hospital, 23071 Jaen, Spain; 6 Biochemical Laboratory, Reina Sofia University Hospital, 14004 Córdoba, Spain

**Keywords:** genetic variants, healthy diets, coronary heart disease, role of sirtuins, aging-related processes

## Abstract

We investigated whether long-term consumption of two healthy diets (low-fat (LF) or Mediterranean (Med)) interacts with *SIRT1* genotypes to modulate aging-related processes such as leucocyte telomere length (LTL), oxidative stress (OxS) and inflammation in patients with coronary heart disease (CHD). LTL, inflammation, OxS markers (at baseline and after 4 years of follow-up) and *SIRT1*-Single Nucleotide Polymorphisms (SNPs) (rs7069102 and rs1885472) were determined in patients from the CORDIOPREV study. We analyzed the genotype-marker interactions and the effect of diet on these interactions. Regardless of the diet, we observed LTL maintenance in GG-carriers for the rs7069102, in contrast to carriers of the minor C allele, where it decreased after follow-up (*p* = 0.001). The GG-carriers showed an increase in reduced/oxidized glutathione (GSH/GSSG) ratio (*p* = 0.003), lower lipid peroxidation products (LPO) levels (*p* < 0.001) and a greater decrease in tumor necrosis factor-alpha (TNF-α) levels (*p* < 0.001) after follow-up. After the LF diet intervention, the GG-carriers showed stabilization in LTL which was significant compared to the C allele subjects (*p* = 0.037), although the protective effects found for inflammation and OxS markers remained significant after follow-up with the two diets. Patients who are homozygous for the *SIRT1*-SNP rs7069102 (the most common genotype) may benefit from healthy diets, as suggested by improvements in OxS and inflammation in patients with CHD, which may indicate the slowing-down of the aging process and its related diseases.

## 1. Introduction

According to the World Health Organization (WHO), there will be a significant increase over the next decade in the mortality rate from age-related chronic diseases (cardiovascular disease, diabetes, etc.) [1]. The WHO has stated that the majority of the population will have a life expectancy of 60 years or more and predicts that between 2015 and 2050, the percentage of the world’s population aged 60 years old and over will almost double from 12% to 22% [2]. 

Many different factors are involved in cellular aging, such as an increase in oxidative stress (OxS) and inflammation, which impacts the functionality of molecules or structures related to aging, such as telomere length, among others. Previous results by our research group have demonstrated that a low intake of vitamin E accelerates cellular aging in patients with cardiovascular disease [3]. This suggests the need to focus studies on identifying patients at risk of unhealthy aging to take more effective and powerful steps aimed at delaying or slowing the aging process, and thus reducing the probability of developing aging-related diseases.

A healthy lifestyle, including a healthy diet, improves the maintenance of telomere length, inflammatory and OxS status, and adherence to an unhealthy lifestyle has the opposite effect [4]. Among the healthy diets that have been linked to an improvement in age-related changes are the Mediterranean (Med) diet [5,6,7] and low-fat (LF) dietary patterns [8,9,10]. 

There is a genetic component in aging and in age-related diseases [11,12] where single nucleotide polymorphisms (SNPs) have been shown to be associated with processes related to the development of these diseases [13,14,15].

Sirtuins are a group of proteins involved in the protection and repair of DNA, as they participate in the deacetylation of histones, molecules that maintain the structure of the genetic sequence, and their dysfunction may be associated with cell damage, senescence or cellular aging [16]. They play a role in preventing OxS and inflammation [17] and are present in various intracellular locations in different tissues (liver, adipose tissue, endothelium, etc.). Their functionality is regulated by external factors such as diet [18,19], among others. For this reason, previous studies have focused on the regulation by sirtuins of the aging process and the development of age-related diseases [20,21]. The *SIRT1* gene encodes for the protein sirtuin 1 (Sirt1), which has a range of molecular functions and has emerged as an important protein in aging and metabolic regulation [22]. During the past decade, studies have suggested a correlation between Sirt1 activity and aging-associated diseases, including diabetes, cardiovascular disease and neurodegenerative disorders [23]. SNPs rs7069102 and rs1885472 are two SNPs in high LD located in the gene sequence. In a previous study with healthy people, the risk of developing cardiovascular disease was associated with the rs7069102 genotype, which points to the relationship between that polymorphism and cardiovascular disease [24]. However, to date, no research has looked into whether the biological effects of these SNPs are partly related to their association with age-related features. 

Based on the above, we investigated whether long-term consumption of two healthy dietary patterns (LF diet or Med diet) interact with genetic variability at the *SIRT1* gene locus to modulate aging-related processes such as telomeres length, OxS and inflammation in patients with coronary heart disease (CHD).

## 2. Materials and Methods

### 2.1. Population

The current work was conducted within the framework of the CORDIOPREV study. The CORDIOPREV study was a prospective, randomized, controlled clinical trial. Dietary intervention was performed in patients with CHD whose inclusion period began in 2009. It included a total of 1002 patients between the ages of 20 and 75 years old, who took part in a dietary intervention with a Med or LF diet for a median of seven years. The details of the study design have been previously provided on Clinicaltrials.gov (NCT00924937) and the inclusion and exclusion criteria have been previously described [5]. Written consent was obtained from all the subjects before recruitment and the study protocol and all amendments were approved by the Ethics Committee of Hospital Reina Sofia, all of which follow the Helsinki Declaration and good clinical practices. We carried out our analysis in those subjects for whom we had complete information on the variables studied in the baseline situation and after 4 years of follow-up (clinical, biochemical, genetic and telomere length data (*n* = 716). 

### 2.2. Diet, Dietary Assessment and Follow-Up Visits

The patients were randomized into two different healthy dietary patterns: a Med diet rich in fat from olive oil, with >35% of calories from fat (22% monounsaturated (MUFA), 6% polyunsaturated (PUFA), <10% saturated (SFA)), 15% proteins and a maximum of 50% carbohydrates; and a LF diet, comprising <30% total fat (12–14% MUFA, 6–8% PUFA and <10% SFA), 15% protein, and a minimum of 55% carbohydrates. Briefing sessions were held for each type of diet and a regular follow-up was carried out by nutritionists, as detailed in previous publications of our group [25]. Dietary adherence in the CORDIOPREV study has previously been reported by Quintana-Navarro et al. [25]. Full study diets, dietary assessments, and follow-up visits have previously been reported [26].

### 2.3. Laboratory Measurements

Details of the measurements have been provided in a previous publication [27]. Blood samples were collected from the participants after a 12 h overnight fast at the beginning of the study and once a year during the follow-up period. In the present work, we used the information about biochemical parameters obtained at the beginning of the study and after 4 years of follow-up. Plasma and serum samples were collected in individual tubes and centrifuged, then immediately frozen at −80 °C. The biochemical measurements were taken at the Reina Sofia University Hospital by staff who had no knowledge of the interventions. The main biochemical variables used in the present study were triglycerides, total cholesterol, high density lipoprotein cholesterol (HDL-c), low density lipoprotein cholesterol (LDL-c), glucose and C- reactive protein (CRP). The methodologies used to carry out the measurements have been described previously [27].

### 2.4. OxS- and Inflammation-Related Parameters

As a sample of the total population of the study, oxidative-stress and inflammation-related data from 353 participants from the CORDIOPREV Study were acquired in the set of the PI13-00185 Grant (see acknowledgments), as previously published by our group [28]. Lipid peroxidation products (LPO), total glutathione, reduced (GSH) and oxidized glutathione (GSSG) and the GSH/GSSG ratio were determined, as previously defined [28]. GSH and GSSG content were measured in the plasma samples using the BIOXYTECH^®^ GSH-400 Kit (OXIS International Inc., Portland, OR, USA) and the GSH-412 Kit (OXIS International Inc., Portland, OR, USA), respectively. Tumor necrosis factor-alpha (TNF-α) was measured using commercially available enzyme-linked immunosorbent assay ELISA kits (R & D Systems, Minneapolis, MN, USA). 

### 2.5. DNA Isolation from Blood Samples

DNA was obtained from the blood cells of the buffy coat fraction from blood contained in EDTA tubes. The DNA was isolated through the salting-out method, and the DNA was then resuspended in 500 μL of 1 × TE buffer [29]. 

### 2.6. Genotyping

Genotyping was performed using the OpenArray™ platform provided by Thermo Fisher Scientific Inc. (Waltham, MA, USA), using TaqMan assays C___1340389_10 (assay name: hCV1340389) for rs7069102 and C___11642237_10 (assay name: hCV11642237) for rs1885472 provided by Thermo Fisher Scientific Inc. (Database: https://www.thermofisher.com/es/es/home/life-science/pcr/real-time-pcr/real-time-pcr-assays/snp-genotyping-taqman-assays.html) (accessed on 13 September 2020). The procedure was carried out according to the manufacturer’s instructions using an AccuFill™ robot for array loading, a 9700 thermal cycler for polymerase chain reaction (PCR) and an NT cycler for fluorescence reading (all provided by Thermo Fisher Scientific Inc.). The Hardy–Weinberg equilibrium was measured using the gene calc bioinformatic tool [30]. TaqMan™ Genotyper software V 1.3 (Life Technologies, Carlsbad, CA, USA) was used for genotype calling. We used 1000 GENOMES phase_3:IBS (http://www.ensembl.org/Homo_sapiens/Info/Index) (accessed on 13 September 2020), as the reference population. The Pairwise LD level among *SIRT1* SNPs was validated by analyzing the D′ and r^2^ values using Ensembl (Human GRCh38.p13) in the same population group.

### 2.7. Quantitative PCR Analysis of Leucocyte Telomere Length (LTL)

We analyzed LTL at the beginning and after four years of follow-up, as per the study protocol. We used the Cawthon method with quantitative PCR [31], as in the previous papers from our group [27,32,33]. PCR reactions were carried out on an iQ5 thermal cycler using a SensiFAST™ SYBR Lo-ROX kit, where for all samples the ratio of telomere to constitutive gene RPL13a was estimated. The sequence of primers used and PCR conditions have previously been published [27].

### 2.8. Statistics

We used SPSS Statistics for Windows (version 28.0) (IBM, Chicago, IL, USA) and data are presented as mean ± standard error of the mean (SEM). The differences in mean biochemical and anthropometric parameters and the interaction with the genotype were evaluated by One-Way ANOVA analysis (Supplementary Table S1). The comparison of clinical and anthropometric parameters according to diet and genotype was carried out using One-Way ANOVA analysis, separately and independently for each diet, with the SNP rs7069102 genotype as a factor. Comparisons of frequencies between qualitative variables were carried out using the Chi-Square test. ANOVA for repeated measures was used to analyze the changes during the study. The Greenhouse-Geisser contrast statistic was used when the sphericity assumption was not satisfied. In this analysis, we studied the overall genotype influence (global ANOVA, *p* for genotype influence), the kinetics of the intervention (*p* for time), and the interaction of the two factors (genotype vs. time). When post hoc test analyses were pertinent, we used multiple comparison tests with the Sidak correction. *p* < 0.05 was considered statistically significant.

## 3. Results

### 3.1. Baseline Characteristics according to the SIRT1 SNPs Genotype

The genotype distributions of both SNPs did not deviate from the Hardy–Weinberg expectations. We compared the allelic frequencies observed for both *SIRT1* SNPs with the 1000 genomes database. The minor allele frequency for the SNP rs7069102 was C = 0.305 (1000 genomes: C = 0.355) and for the SNP rs1885472 was G = 0.317 (1000 genomes: G = 0.355). The genotypic frequencies observed for the SNP rs7069102 were G/G = 47.78%; G/C = 43.43%; C/C = 8.79%, and for the SNP rs1885472 were C/C = 47.02%; C/G = 42.45%; G/G = 10.53%. The LD analysis showed that the SNPs rs1885472 and rs7069102 were D′ = 1.000 and r^2^ = 1.000, suggesting that the loci are in complete LD and coinherited in most cases. Therefore, given its association, and the existence of previous literature with SNP rs7069102, but not with rs1885472, we decided to continue showing the analysis with rs7069102 in this article. Since our analyses did not support a recessive mode of action for this SNP and given the low frequencies of the genotype CC in rs7069102, we conducted our subsequent analyses using a dominant model for the minor allele. 

The demographic, anthropometric and biochemical characteristics according to the *SIRT1* SNP rs7069102 and diet are presented in Table 1. At baseline, we observed a significant difference in weight in patients randomized to the Med diet who were also carriers of the C allele compared to carriers of the GG genotype (*p* = 0.043). No significant differences were observed in the other parameters analyzed, nor in the patients who followed the LF dietary pattern (Table 1). No significant differences according to genotype for baseline anthropometrics, cholesterol, glucose or CRP values were observed for the *SIRT1* SNP rs7069102 (Supplementary Table S1).

### 3.2. Relationship between the SNP rs7069102 and LTL

**Figure 1:**
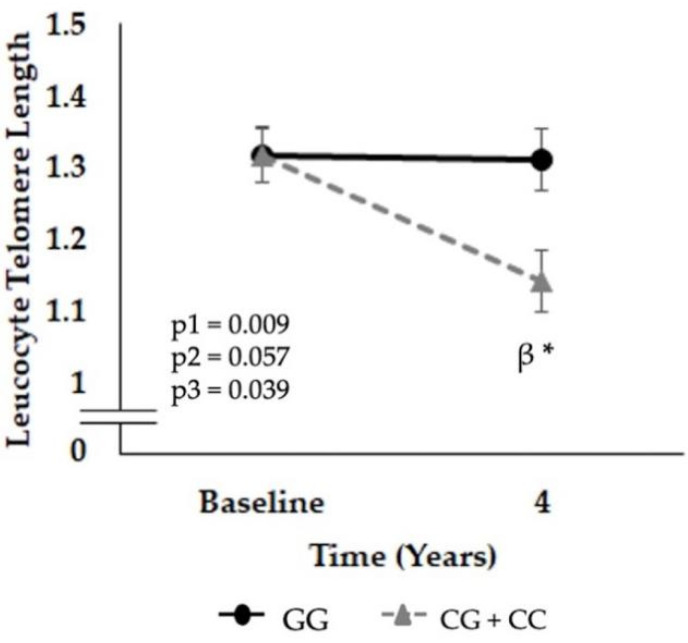
Leucocyte telomere length according to the single nucleotide polymorphism (SNP) rs7069102 genotype located on the *SIRT1* gene after the follow-up period. Data are represented as the mean ± SEM and correspond to ANOVA for repeated measures; where p1: time follow-up, p2: genotype influence and p3: the interaction of the two factors (time vs. genotype). When post hoc tests were pertinent, we used multiple comparisons with the Sidak correction. * *p* < 0.05 4 years vs. baseline, β *p* < 0.05 genotypes in the same time. The data correspond to the population included in the study where 351 subjects are carriers of the GG genotype (49%) and 365 of the CG + CC genotype (51%) (combined both LF and Med diets).

In the whole population, we observed a maintenance of LTL in patients carrying the GG genotype, in contrast to carriers of the mutant C allele, in whom LTL decreased after 4 years of follow-up compared with baseline (*p* = 0.001) (Figure 1).

### 3.3. Relationship between the SNP rs7069102 and Inflammation and OxS-Related Parameters

**Figure 2:**
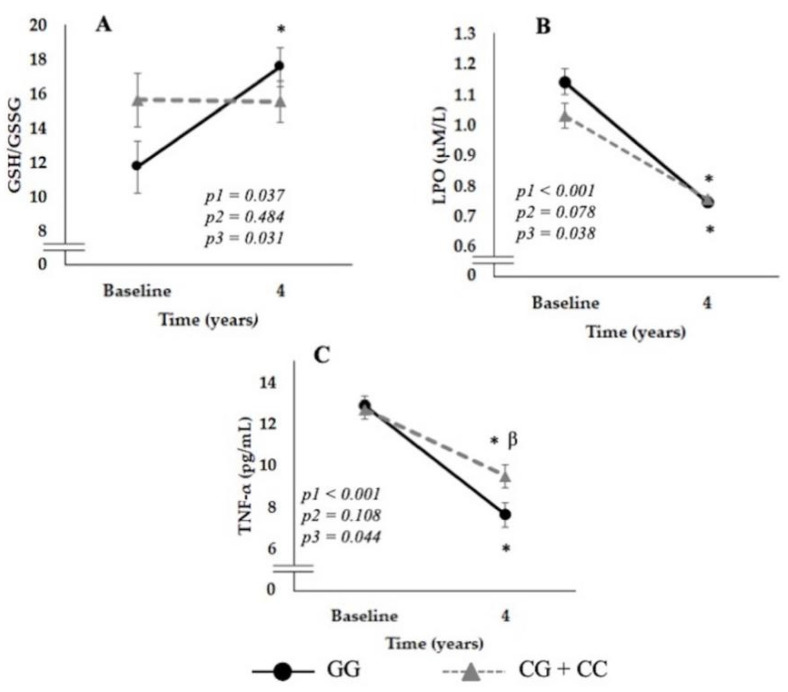
Changes in variables associated with oxidative stress (OxS) and inflammation according to SNP rs7069102 genotype located in *SIRT1* gene (Panel **A**, reduced/oxidized glutathione (GSH/GSSG); **B**, lipid peroxidation products (LPO); **C**, tumor necrosis factor-alpha TNF-α). Data are represented as the mean ± SEM and correspond to ANOVA for repeated measures; where p1: time of follow-up, p2: genotype influence and p3: the interaction of the two factors (time vs. genotype). When post hoc tests were pertinent, we used multiple comparisons with the Sidak correction. * *p* < 0.05 4 years vs. baseline, β *p* < 0.05 genotypes in the same time. The data correspond to the population included in the study selected to the OxS and inflammation analyses where the where 49% carried the GG genotype and 51% carried the C allele.

Subjects with the GG genotype showed an increase in GSH/GSSG ratio values compared to their baseline levels (*p* = 0.003) (Figure 2A). Additionally, the LPO levels decreases in both genotypes (GG and CG + CC) (both *p* < 0.001), however it decreases more in GG carriers after the intervention period compared to baseline (Figure 2B). Moreover, a significant decrease in TNF-α levels was observed in both the GG and CG + CC genotypes (both *p* < 0.001, respectively), although this profile was significantly greater in GG homozygotes after 4 years of follow-up (*p* = 0.022) (Figure 2C).

### 3.4. Effect of Diet on LTL and Inflammation-Related Parameters According to SIRT1 Gene Variants

#### 3.4.1. Patients Randomized to the LF Diet

**Figure 3:**
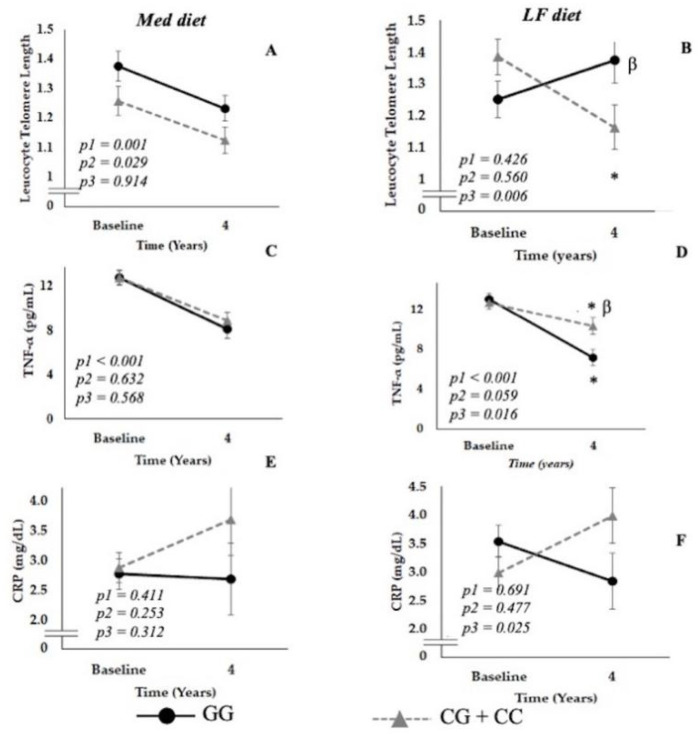
Interaction between diet and aging- and inflammation-related parameters according to SNP rs7069102 genotype located in *SIRT1* gene (Panels **A**,**C**,**E**, Mediterranean Diet and Leukocyte Telomere Length, TNF-α and CRP; Panels **B**,**D**,**F**, Low-Fat Diet and Leucocyte Telomere Length, TNF-α and CRP). Data are represented as the mean ± SEM and correspond to an ANOVA for repeated measures, where p1: time of follow-up, p2: genotype influence and p3: the interaction of the two factors (time vs. genotype). When post hoc tests were pertinent, we used multiple comparisons with the Sidak correction. * *p* < 0.05 4 years vs. baseline, β *p* < 0.05 genotypes in the same time period.

For the SNP rs7069102, after dietary intervention with the LF diet, a stabilization in LTL was observed in subjects with the GG genotype, which decreased significantly in subjects carrying the CG + CC genotypes (*p* = 0.012) (Figure 3B). 

TNF-α levels decreased after 4 years in subjects carrying both genotypes (GG *p* < 0.001 and CG + CC *p* = 0.020). However, this decrease was significantly greater in subjects carrying the GG genotype (*p* = 0.007) (Figure 3D). These GG patients also showed a significant decrease in CRP levels when compared with carriers of the C allele (*p* time vs. genotype = 0.025) (Figure 3F). 

#### 3.4.2. Patients Randomized to Med Diet

Telomere length decreased in GG and GC+CC patients after 4 years of follow-up. No significant differences were observed between GG and GC+CC patients in terms of TNF-α levels and CRP levels (Figure 3A,C,E).

## 4. Discussion

Our results demonstrate the interaction between diet and rs7069102 gene variation of the *SIRT1* gene in the modulation of aging-associated markers. We observed that subjects homozygous for the common allele who followed a LF dietary pattern for 4 years showed, over time, benefits in telomere length evolution, which is a marker of cellular aging. Additionally, in these patients, we also observed a decrease in TNF-α and CRP, which are markers of OxS and inflammation and factors previously associated with telomere length. In parallel, and as an internal control study, we performed the same data analysis with the high LD association rs1885472 that replicated all the findings for rs7069102.

The WHO estimates that deaths from chronic diseases associated with aging will double in the next 10 years. This is because the percentage of the world’s population aged 60 years old and over will increase almost two-fold from 12% to 22% [2]. The development of diseases associated with aging is directly related to cellular senescence, where cells are unable to carry out certain biological processes. In various metabolic diseases such as diabetes mellitus and cardiovascular diseases, the adoption of healthy eating habits has been proven to be useful to slow down the aging process and cellular dysfunction. Previous studies have demonstrated the beneficial effect of adherence to diets such as the Med diet or LF diet on markers associated with aging [10,32]. However, nutritional habits may have a different effect on the aging process, partly due to the genetic diversity of the population. In fact, during the aging process, these genetic characteristics also determine changes at the cellular level such as apoptosis, telomeric shortening and mitochondrial activity [32,34,35]. This directly impacts on markers associated with OxS (GSH and GSSG, LPO), inflammation (TNF-α and CRP) and telomere length.

In this context, the proteins known as sirtuins, due to their involvement in apoptosis, inflammation and mitochondrial activity, are related to the protection against arterial calcification, the regulation of mitochondrial function and the antioxidant defense system [36]. Although the rs7069102 genotype was previously associated with an increase in the risk of developing cardiovascular disease in healthy subjects [24], it was not previously associated with changes in cellular age-related features, such as telomere length. Regarding the other SNP chosen for investigation, rs1885472, there is little previous evidence available about its relationship with aging, disease and how environmental factors such as diet affect it, but its high LD with rs7069102 enabled us to confirm the data obtained. Due to the high co-inheritability, we only showed the results for rs7069102, selecting it as an SNP tag. However, we also performed all the analyses with rs1885472 (data not shown), which replicated the results obtained for rs7069102. The consistency of these results with rs1885472 provides an internal control which helps to corroborate the main body of evidence.

In our study, when we analyzed the whole population, not splitting the sample by diet type, subjects carrying the mutated alleles showed a decrease, after 4 years, in telomere length, in contrast to the maintenance in the telomere length found in the homozygotes for the common allele. Although concomitant improvements in OxS (LPO) and inflammation parameters (TNF-α) were found in all participants, these changes were more favorable in the common allele homozygotes. 

One interesting finding of our study was that telomere length did not differ at the beginning of the study between C-carriers and GG patients. We believe that this is a clear case of Nutrigenomics, rather than a genetic effect, as we were not able to see differences in the telomeres in subjects who were not distributed to different dietary patterns, and that it was precisely the dietary intervention that allowed us to find this association between nutrition and genotype. In this context, in relation to diet, a previous study analyzed the association between *SIRT1* SNPs (rs7069102, rs2273773, rs3818292), suggesting that the combination of genetic variants of the *SIRT1* gene and dietary n-6 and/or n-3 PUFA intake influences the serum levels of LDL-C and HDL-C [37]. In our study, we observed that carriers of the mutated alleles at SNPs rs7069102 (and rs1885472), after a 4-year follow-up of a LF diet, showed some changes in parameters associated with ageing (telomere length) and inflammation, which were not found in carriers of the ancestral alleles. 

As mentioned above, when we analyzed the data from the whole population (without splitting the population into dietary arms), we saw beneficial differences in variables related to aging in the subjects homozygous for the common allele. This could be interpreted as a favorable association following the intervention with either diet. Specific evaluation of the results by diet showed that these effects were only statistically evident in the subgroup of the LF diet, which may indicate that the LF diet performed better, or that we were not able to identify those specific beneficial effects in the Med diet group due to a lack of statistical power for the results obtained. Nevertheless, to provide an explanation underlying our results, we should remember that, although the Med Diet and the LF diet show some common features, there are differences, such as the fat content, that could act as drivers of the associations found. 

Our study has certain limitations. It was conducted in a population with CHD, which suggests that our results should not be extrapolated to the general population. Additionally, in our study, we analyzed telomere length over a certain time period, which does not allow us to infer either the total length of the telomere or the changes that this length had in the years before of the study started, when the subjects were on an ad libitum diet. Finally, larger-scale studies would be needed to replicate our findings. 

## 5. Conclusions

In conclusion, the genetic variants located in the *SIRT1* gene will enable us to offer our patients a personalized diet tailored to their genetic characteristics, which allows us to slow down the aging process and its related diseases by improving the OxS and inflammation-related parameters. In our case, homozygotes for the rs7069102 SNP would benefit from a LF diet to slow telomere shortening. 

## Figures and Tables

**Table 1:** Characteristics of patients according to genotype for *SIRT1* polymorphism rs7069102 and diet.

Parameters	LF Diet			Med Diet		
	GG	CG + CC	*p* Value	GG	CG + CC	*p* Value
N	162	171		189	194	
Men/Women (n)	134/28	145/26	0.607	164/25	151/43	0.022 *
Age (years)	58.7 ± 8.7	59.6 ± 8.1	0.329	59.3 ± 8.9	59.8 ± 9.2	0.629
Weight (kg)	85.3 ± 12.8	84.5 ± 12.2	0.547	86.6 ± 14.2	83.7 ± 14	0.043 *
Waist circumference (cm)	104.5 ± 10.4	105.1 ± 10.2	0.628	105.2 ± 11.5	104.1 ± 11	0.311
BMI (kg/m^2^)	31.2 ± 4.3	30.7 ± 4	0.289	31.4 ± 4.4	30.9 ± 4.4	0.315
Total cholesterol (mg/dL)	157.2± 29.8	160.1 ± 28.2	0.359	158.5 ± 33.1	159.6 ± 31.5	0.723
HDL-C (mg/dL)	42.1 ± 10	42.9 ± 10.1	0.452	42.2 ± 10.3	42.1 ± 10.3	0.931
LDL-C (mg/dL)	86.8 ± 23.9	89.7± 23.5	0.281	88.2 ± 26.6	90.1 ± 25.4	0.486
TG (mg/dL)	128 ± 64.4	142.2 ± 73.4	0.063	137.1 ± 71	130.1 ± 64.5	0.315
Glucose (mg/dL)	108.8 ± 29.5	113.4 ± 38.3	0.219	115.4 ± 40.8	111.4 ± 36.3	0.316
CRP (mg/dL)	3.6 ± 4.3	2.8 ± 3.2	0.073	2.7 ± 3.7	2.8 ± 3.1	0.945

Values are expressed as mean ± SEM. LF, low fat; Med, Mediterranean; BMI, Body mass index; HDL-C, high-density lipoprotein; LDL-C, low-density lipoprotein; TG, triglycerides; CRP, C-reactive protein. Variables were calculated using One-Way ANOVA analysis. * *p* values < 0.05.

## Data Availability

The data presented in this study are available in the repositories of the Maimónides Biomedical Research Institute of Córdoba (IMIBIC), upon request to the corresponding authors and to the principal investigators of the project P.P.-M., J.D.-L. and J.L.-M.

## Supplementary Materials

The following supporting information can be downloaded at: https://www.mdpi.com/article/10.3390/nu14183789/s1, Table S1. Characteristics of patients included in the study according to the genotype for the *SIRT1* polymorphism rs7069102.
