# Supplementary material for: Diet and SIRT1 Genotype Interact to Modulate Aging-Related Processes in Patients with Coronary Heart Disease: From the CORDIOPREV Study
Source: Nutrients. 2022 Sep 14;14(18):3789. doi: 10.3390/nu14183789 (PMC9504765; doi:10.3390/nu14183789)
Supplement: Supplementary file 1 [file nutrients-14-03789-s001.zip › nutrients-1887384-supplementary.pdf]

Table S1. Characteristics of patients included in the study according the genotype for the SIRT1 polymorphism rs7069102.

	rs7069102		
	**GG**	**CG+CC**	**p**
N	351	365	
Men/women (n)	298/53	296/69	0.176
Age (years)	59 ± 8.8	59.7 ± 8.7	0.316
Weight (kg)	86 ± 13.6	84.1 ±13.2	0.052
Waist circunference (cm)	104.9 ± 11	104.5 ± 10.6	0.649
BMI (kg/m^2)	31.3 ± 4.4	30.8 ± 4.3	0.144
Total colesterol (mg/dL)	157.9 ± 31.6	159.9 ± 30	0.389
HDL-C (mg/dL)	42.1 ± 10.2	42.5 ± 10.2	0.652
LDL-C (mg/dL)	87.6 ± 25.4	89.9 ± 24.5	0.220
TG (mg/dL)	132.9 ± 68.1	135.8 ± 69.1	0.573
Glucose (mg/dL)	112.3 ± 36.2	112.4 ± 37.2	0.995
CRP (mg/dL)	3.1 ± 4	2.8 ± 3.1	0.229

Values expressed as mean ± SEM. BMI, Body mass index; HDL-C, high density lipoprotein; LDL-C, low density lipoprotein; TG, triglycerides; CRP, C-reactive protein. Variables were calculated using One-Way ANOVA analysis.
